# Midazolam as a Sedative in Agitated or Aggressive Patients Before Psychiatric Admission

**DOI:** 10.1192/j.eurpsy.2025.1452

**Published:** 2025-08-26

**Authors:** V. Anagnostopoulou, P. Argitis, M. Demetriou, M. Peyioti, Z. Chaviaras

**Affiliations:** 1 Psychiatry Department, General Hospital of Corfu, Corfu; 2 University General Hospital of Alexandroupolis, Alexandroupolis, Greece

## Abstract

**Introduction:**

Managing agitated or aggressive patients prior to psychiatric admission is a critical challenge in clinical practice. When verbal de-escalation fails, effective pharmacological interventions, including benzodiazepines and neuroleptics, are necessary, either orally or via injectable forms. In some cases, physical restraint is inevitable, which is challenging and distressing for both patients and staff members Midazolam, a short-acting benzodiazepine, is well-known for its rapid sedative effects and potential to minimize the need for physical restraint in such patients.

**Objectives:**

To evaluate the efficacy of oral midazolam in providing rapid sedation in agitated patients, facilitating their pre-admission management to psychiatric units.

**Methods:**

A review of clinical practices and relevant studies was conducted to assess the use of midazolam for sedation in patients displaying aggression or agitation prior their admission to a psychiatric unit. The evaluation focused on the drug’s pharmacological profile, including its onset of action, duration, and impact on patient care and staff workflow.

**Results:**

Oral midazolam, due to its rapid onset and short duration of action, effectively provides sedation in acutely agitated patients. Its use significantly reduces the need for physical restraints or other invasive interventions, improving patient comfort and making the admission process smoother for the healthcare staff.

**Conclusions:**

Midazolam presents a promising pharmacological option for pre-admission sedation of agitated or aggressive patients in psychiatric settings. Its rapid sedative effect, combined with its short duration, enhances patient management and minimizes the need for physical restraints, ultimately helping both patients and staff. Further research is needed to standardize its use in psychiatric emergencies.

**Keywords:**

Midazolam, sedation, agitation, psychiatric admission, aggression, pre-admission management.

**Disclosure of Interest:**

None Declared

